# Poly[3-methyl­pyridinium [(μ_2_-di­hydrogen phosphito)bis(μ_3_-hydrogen phosphito)dizinc]]

**DOI:** 10.1107/S2414314624003456

**Published:** 2024-04-26

**Authors:** Jago G. Love-Jennings, Aidan P. McKay, David B. Cordes, William T. A. Harrison

**Affiliations:** a Department of Chemistry, University of Aberdeen, Meston Walk, Aberdeen AB24 3UE, Scotland, United Kingdom; b EaStCHEM, School of Chemistry, University of St Andrews, St Andrews KY16 9ST, Scotland, United Kingdom; Sunway University, Malaysia

**Keywords:** crystal structure, zincophosphite, layered structure, bifurcated hydrogen bond

## Abstract

In the title compound, {(C_6_H_8_N)[Zn_2_(HPO_3_)_2_(H_2_PO_3_)]}_*n*_, the constituent ZnO_4_, HPO_3_ and H_2_PO_3_ polyhedra of the inorganic component are linked into (010) sheets by Zn—O—P bonds (mean angle = 134.4°) and the layers are reinforced by O—H⋯O hydrogen bonds. The protonated templates are anchored to the inorganic sheets *via* bifurcated N—H⋯(O,O) hydrogen bonds.

## Abstract 2

The title compound features bifurcated template-to-framework N—H⋯(O,O) hydrogen bonds.

## Graphical abstract

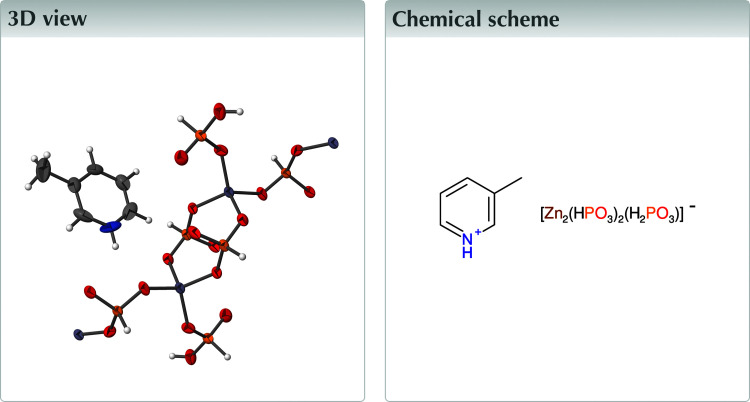

## Structure description

The family of zincophosphite (ZnPO) networks templated or ligated by organic species now encompasses well over 200 crystal structures in the Cambridge Structural Database (CSD; Groom *et al.*, 2016 ▸). In continuation of our ongoing studies of these systems (Holmes *et al.*, 2018 ▸; Wark *et al.*, 2023 ▸), we describe the synthesis and structure of the title compound, {(C_6_H_8_N)[Zn_2_(HPO_3_)_2_(H_2_PO_3_)]}_*n*_, (I), where C_6_H_8_N^+^ is the 3-picolinium (or 3-methyl­pyridinium) cation.

**Figure 1:**
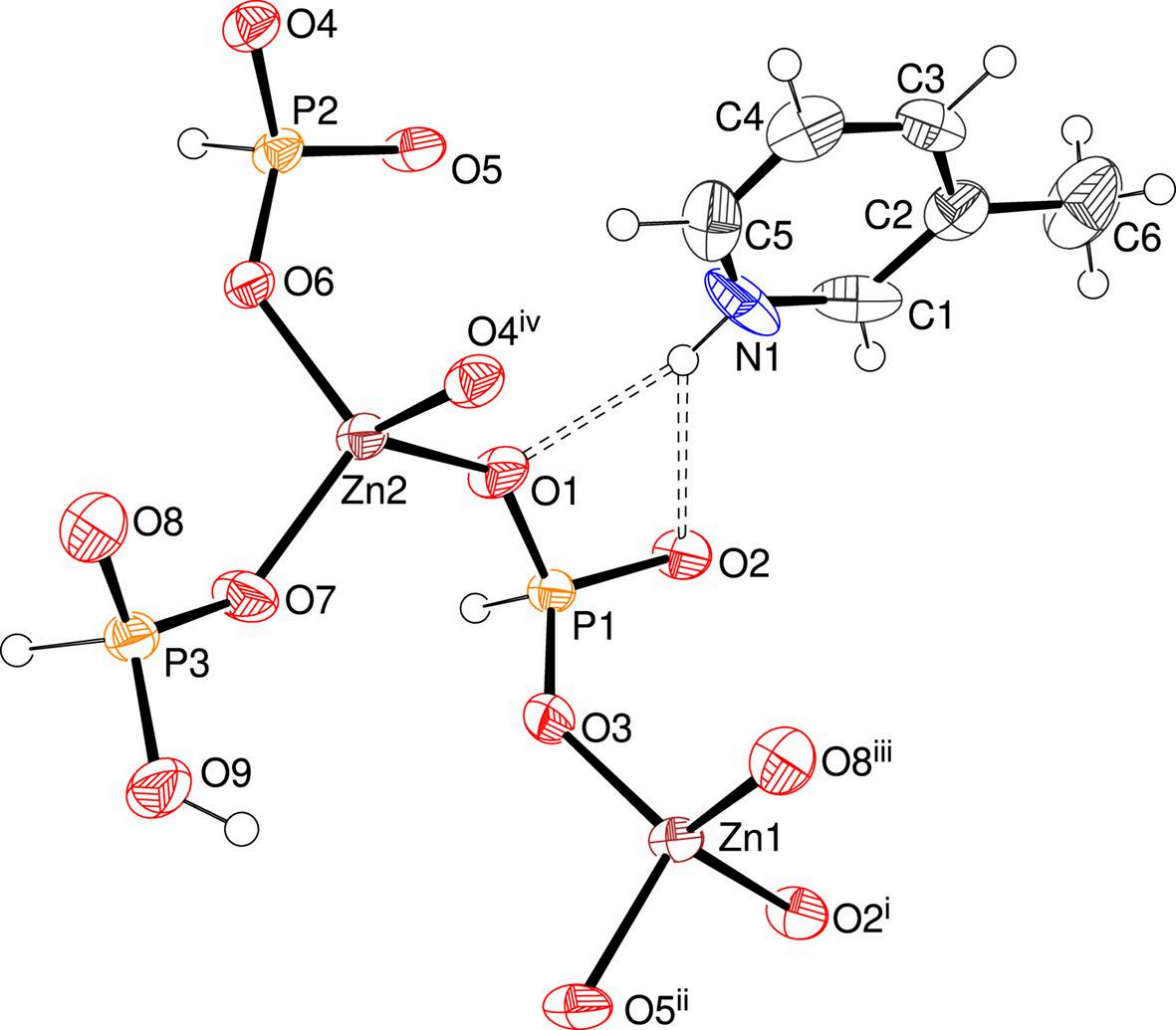
The asymmetric unit of (I), expanded to show the complete zinc-atom coordination spheres, showing 50% displacement ellipsoids. [Symmetry codes: (i) −*x*, −*y* + 1, −*z*; (ii) *x* − 1, *y*, *z*; (iii) −*x*, −*y* + 1, −*z* + 1; (iv) −*x* + 1, −*y* + 1, −*z* + 1.] Hydrogen bonds are indicated by double-dashed lines.

The asymmetric unit of (I) (Fig. 1 ▸), which crystallizes in the triclinic space group *P* 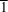 , consists of two Zn^2+^ ions, two [HPO_3_]^2−^ hydrogen phosphite anions, one [H_2_PO_3_]^−^ di­hydrogen phosphite anion and one C_7_H_8_N^+^ cation. The zinc coordination polyhedra are ZnO_4_ tetra­hedra, with mean Zn—O separations of 1.934 and 1.942 Å for Zn1 and Zn2, respectively. The spread of bond angles about the metal ions [100.45 (13)–114.37 (14)° for Zn1 and 102.86 (14)–112.73 (14)° for Zn2] indicate modest degrees of distortion, with τ_4_′ values (Okuniewski *et al.*, 2015 ▸) of 0.95 (Zn1) and 0.96 (Zn2), where a value of 1.00 corresponds to a regular tetra­hedron. The [HPO_3_]^2−^ groups adopt their usual tetra­hedral (including the H atom) or pseudo-pyramidal (excluding H) shape and the mean P—O separations are 1.506 Å for P1 and 1.516 Å for P2. The O—P—O bond angles around P1 show a larger than typical range of 107.52 (19)–114.3 (2)°, with the smallest O1—P1—O2 angle associated with the bifurcated hydrogen bond from the protonated template (Fig. 1 ▸), whereas the P2 bond angles are tightly clustered [112.38 (19)–112.74 (18)°]. The [H_2_PO_3_]^−^ di­hydrogen phosphite group containing atom P3 includes a notably longer vertex [P3—O9 = 1.543 (3) Å] to the protonated O atom. Apart from O9, each O atom in (I) is bonded to one Zn and one P atom: the Zn—O—P bond angles vary from 128.89 (18) to 138.6 (2)°, with a mean of 134.4°, which is typical for this class of material (Wark *et al.*, 2023 ▸). The geometrical parameters for the organic cation are as expected (*e.g.* Sivakumar *et al.*, 2016 ▸).

**Figure 2:**
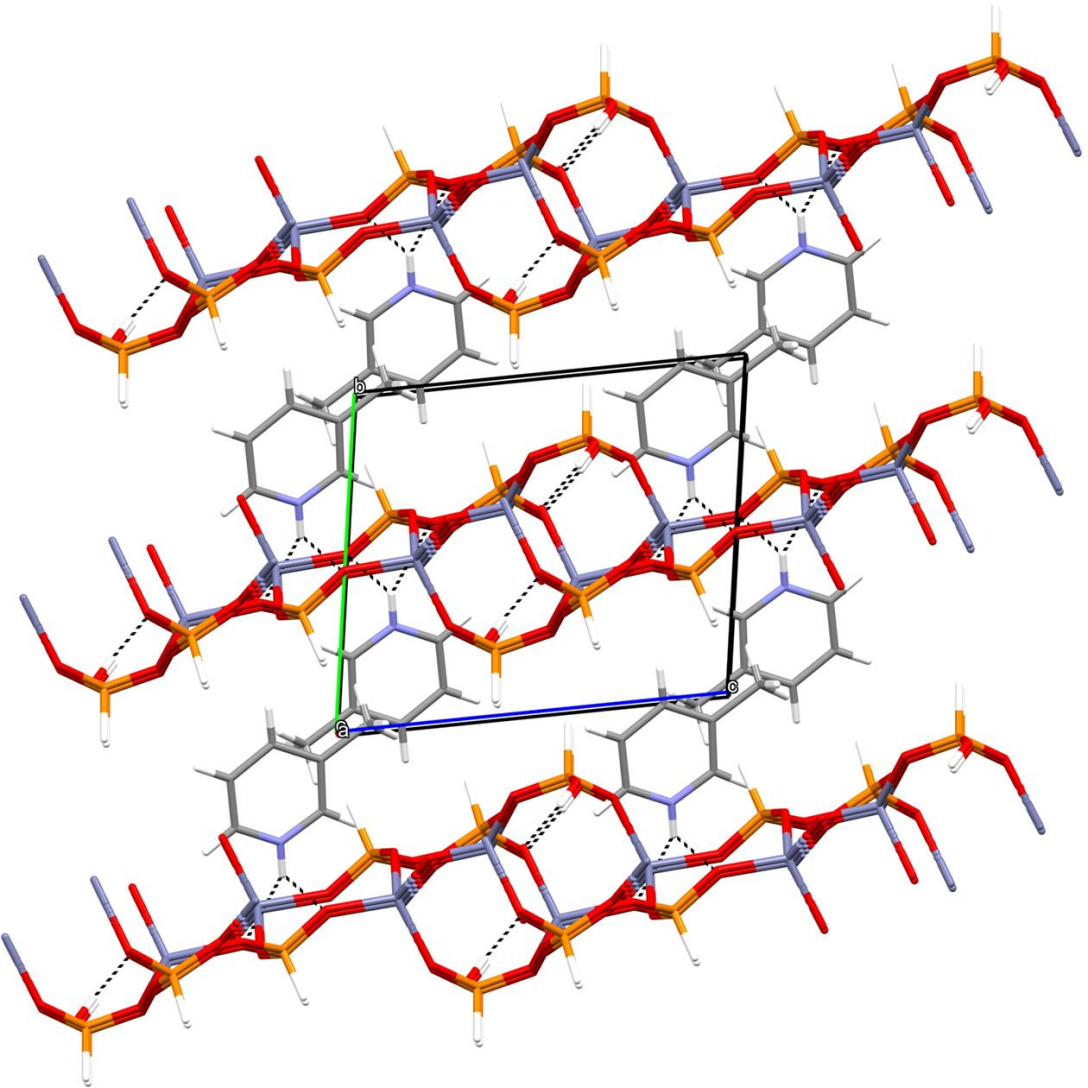
The unit-cell packing in (I), viewed down [100]. Hydrogen bonds are shown as dashed lines.

**Figure 3:**
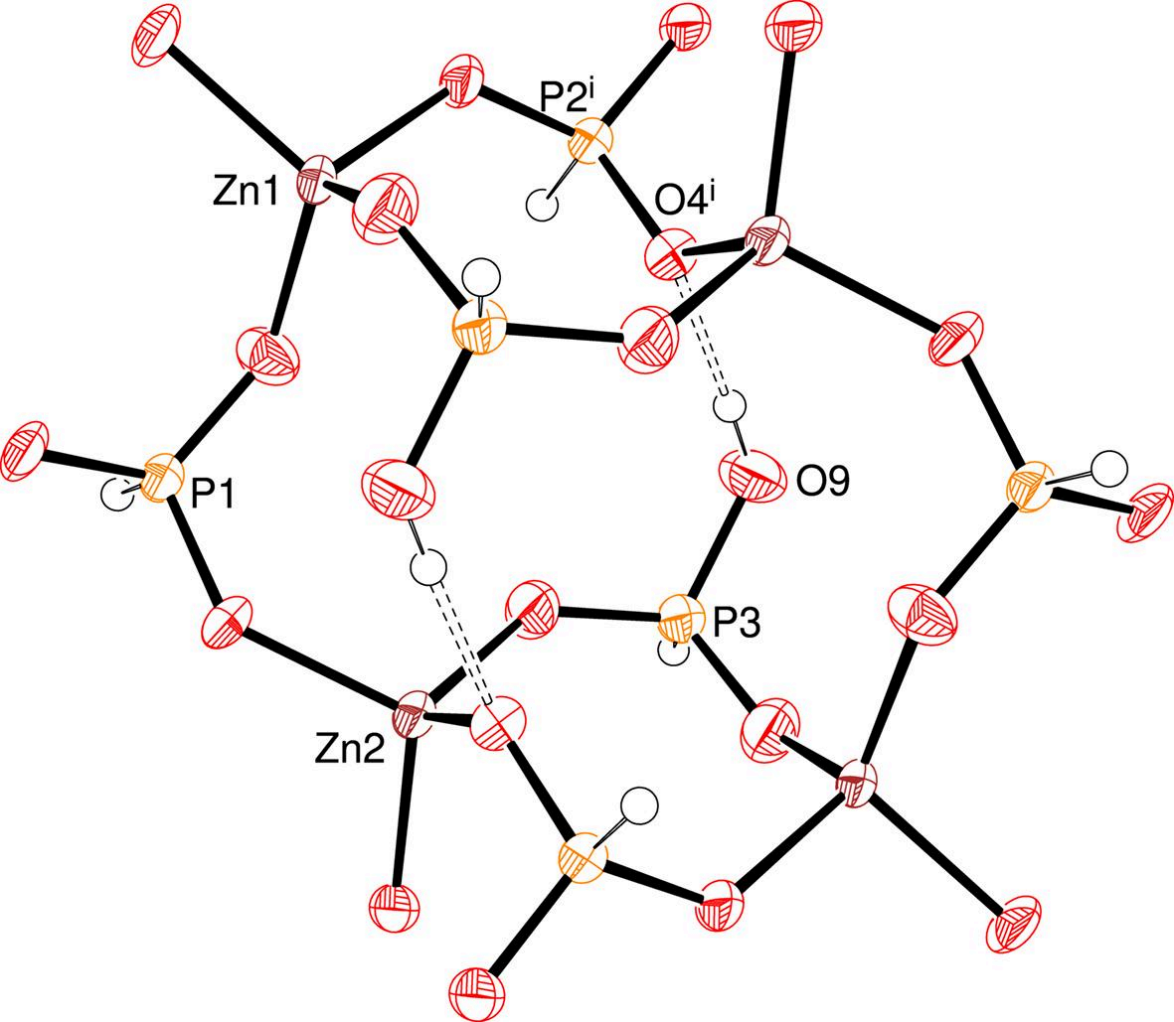
Detail of an infinite (010) polyhedral layer in (I), showing the bifurcated 8-ring reinforced by pairwise O—H⋯O hydrogen bonds (double-dashed lines). [Symmetry code: (i) *x* − 1, *y*, *z*.]

In the extended structure of (I), the constituent ZnO_4_, HPO_3_ and H_2_PO_3_ polyhedra are linked by Zn—O—P bonds into infinite (010) sheets (Fig. 2 ▸). Polyhedral 4- and 8-rings are present and the zinc and phospho­rus nodes strictly alternate. The most distinctive building unit is a centrosymmetric 8-ring incorporating two bifurcated 4-rings reinforced by a pair of O9—H1O⋯O4 intra-layer hydrogen bonds (Fig. 3 ▸). These are linked by 4-rings involving the Zn2—O6—P2 bonds into [100] chains and crosslinked in the [001] direction by Zn1—O2—P1 bonds into the (010) sheets. The template inter­acts with the inorganic layers *via* an unusual bifurcated N1—H1*B*⋯(O1,O2) link (Fig. 1 ▸): the vast majority of template-to-framework hydrogen bonds are associated with a single acceptor O atom. Some weak nonclassical C—H⋯O inter­actions occur, as listed in Table 1 ▸. As is normal, the P—H unit does not participate in hydrogen bonding (Katinaitė & Harrison, 2017 ▸). There are no aromatic π–π stacking inter­actions in (I)>, the shortest centroid–centroid separation being greater than 5.68 Å, and inter-layer cohesion must be largely due to van der Waals forces.

A survey of the Cambridge Structural Database (Groom *et al.*, 2016 ▸; updated to April 2024) revealed 217 crystal structures containing zinc cations and hydrogen phosphite anions based on a search for a Zn—O—P—H fragment. Structures containing zinc and a di­hydrogen phosphite unit are uncommon with just three examples found, *viz*. bis­(μ_2_-hydrogen phosphito-*O*,*O*′)(hydrogen phosphito-*O*)(2,2′-bi­pyrid­yl)zinc(II) (CSD refocde BEJHUU; Lin *et al.*, 2003 ▸), bis­(μ_2_-hydrogen phosphito-*O*,*O*′)(hydrogen phosphito-*O*)(4,4′-dimethyl-2,2′-bipyrid­yl)dizinc(II) (GICCOL; Lin *et al.*, 2007 ▸) and *catena*-[1-azonio-4-aza­bicyclo­[2.2.2]octane tris­(μ_3_-hydrogen phosphito)(μ_2_-hydrogen phosphito)(1,4-di­aza­bi­cyclo­[2.2.2]octane-*N*)trizinc(II)] (XIZJEW; Liu *et al.*, 2008 ▸). Compounds BEJHUU and GICCOL are closely related ‘zero-dimensional’ bimetallic clusters with bulky chelating ligands, while XIZJEW features the organic species acting both as a ligand (*via* a Zn—N bond) and a protonated template.

## Synthesis and crystallization

Compound (I) was prepared by mixing 0.41 g of ZnO, 0.82 g of H_3_PO_3_ and 0.47 g of 3-picoline (Zn:P:template molar ratio ≃ 1:2:1), which were placed in a 50 ml polypropyl­ene bottle with 20 ml of water and shaken well to result in a white slurry. The bottle was placed in a 353 K oven for 48 h and then removed and allowed to cool to room temperature over about 2 h. The solids were recovered by vacuum filtration to result in a mass of rod-like colourless crystals accompanied by some white solids. IR (diamond window): 3400–2800 cm^−1^ (O—H, N—H stretch), 2450 cm^−1^ (P—H stretch; Ma *et al.*, 2007 ▸).

## Refinement

Crystal data, data collection and structure refinement details are summarized in Table 2 ▸. The O-bound H atom was located in a difference map and refined as riding in its as-found relative location. The P-, N- and C-bound H atoms were located geometrically (P—H = 1.32, N—H = 0.88 and C—H = 0.95–0.98 Å) and refined as riding atoms. The methyl group was allowed to rotate, but not to tip, to best fit the electron density. The constraint *U*_iso_(H) = 1.2*U*_eq_(N, O or P) or 1.5*U*_eq_(methyl C) was applied in all cases. Two peaks greater than 1 e Å^−3^ were found in the final difference map for (I) in the vicinity of the Zn atoms, but they did not correspond to plausible chemical features.

## Supplementary Material

Crystal structure: contains datablock(s) I, global. DOI: 10.1107/S2414314624003456/tk4104sup1.cif

Structure factors: contains datablock(s) I. DOI: 10.1107/S2414314624003456/tk4104Isup2.hkl

CCDC reference: 2349240

Additional supporting information:  crystallographic information; 3D view; checkCIF report

## Figures and Tables

**Table 1:** Hydrogen-bond geometry (Å, °)

*D*—H⋯*A*	*D*—H	H⋯*A*	*D*⋯*A*	*D*—H⋯*A*
N1—H1*B*⋯O1	0.88	2.20	2.992 (6)	150
N1—H1*B*⋯O2	0.88	2.24	2.947 (6)	138
O9—H1O⋯O4^i^	0.85	1.78	2.630 (4)	177
C1—H1*A*⋯O5^ii^	0.95	2.34	3.285 (6)	175
C5—H5⋯O4^iii^	0.95	2.59	3.368 (6)	140
C5—H5⋯O6^iii^	0.95	2.52	3.300 (7)	140

Symmetry codes: (i) 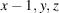 ; (ii) 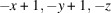 ; (iii) 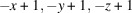 .

**Table 2:** Experimental details

Crystal data	
Chemical formula	(C_6_H_8_N)[Zn_2_(HPO_3_)_2_(H_2_PO_3_)]
*M*_r_	465.82
Crystal system, space group	Triclinic, *P* 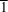
Temperature (K)	173
*a*, *b*, *c* (Å)	8.8428 (5), 9.2779 (6), 9.9343 (4)
α, β, γ (°)	79.126 (4), 82.732 (4), 67.279 (6)
*V* (Å^3^)	736.99 (8)
*Z*	2
Radiation type	Mo *K*α
μ (mm^−1^)	3.62
Crystal size (mm)	0.12 × 0.03 × 0.01

Data collection	
Diffractometer	Rigaku XtaLAB P200K
Absorption correction	Multi-scan (*CrysAlis PRO*; Rigaku OD, 2024 ▸)
*T*_min_, *T*_max_	0.850, 1.000
No. of measured, independent and observed [*I* > 2σ(*I*)] reflections	14787, 3442, 2491
*R*_int_	0.066
(sin θ/λ)_max_ (Å^−1^)	0.695

Refinement	
*R*[*F* ^2^ > 2σ(*F* ^2^)], *wR*(*F* ^2^), *S*	0.050, 0.134, 1.02
No. of reflections	3442
No. of parameters	191
H-atom treatment	H-atom parameters constrained
Δρ_max_, Δρ_min_ (e Å^−3^)	1.43, −0.89

Computer programs: *CrysAlis PRO* (Rigaku OD, 2024 ▸), *SHELXT* (Sheldrick, 2015*a* ▸), *SHELXL2019* (Sheldrick, 2015*b* ▸), *ORTEP-3* (Farrugia, 2012 ▸) and *publCIF* (Westrip, 2010 ▸).

## full crystallographic data

### Crystal data

**Table:**

(C_6_H_8_N)[Zn_2_(HPO_3_)_2_(H_2_PO_3_)]	*Z* = 2
*M**_r_* = 465.82	*F*(000) = 464
Triclinic, *P*1	*D*_x_ = 2.099 Mg m^−^^3^
*a* = 8.8428 (5) Å	Mo *K*α radiation, λ = 0.71073 Å
*b* = 9.2779 (6) Å	Cell parameters from 6732 reflections
*c* = 9.9343 (4) Å	θ = 2.1–29.4°
α = 79.126 (4)°	µ = 3.62 mm^−^^1^
β = 82.732 (4)°	*T* = 173 K
γ = 67.279 (6)°	Bar, colourless
*V* = 736.99 (8) Å^3^	0.12 × 0.03 × 0.01 mm

### Data collection

**Table:**

Rigaku XtaLAB P200K diffractometer	2491 reflections with *I* > 2σ(*I*)
Radiation source: Rotating Anode	*R*_int_ = 0.066
ω scans	θ_max_ = 29.6°, θ_min_ = 2.1°
Absorption correction: multi-scan (CrysAlis PRO; Rigaku OD, 2024)	*h* = −11→12
*T*_min_ = 0.850, *T*_max_ = 1.000	*k* = −12→12
14787 measured reflections	*l* = −13→12
3442 independent reflections	

### Refinement

**Table:**

Refinement on *F*^2^	Primary atom site location: dual
Least-squares matrix: full	Hydrogen site location: mixed
*R*[*F*^2^ > 2σ(*F*^2^)] = 0.050	H-atom parameters constrained
*wR*(*F*^2^) = 0.134	*w* = 1/[σ^2^(*F*_o_^2^) + (0.0819*P*)^2^] where *P* = (*F*_o_^2^ + 2*F*_c_^2^)/3
*S* = 1.02	(Δ/σ)_max_ < 0.001
3442 reflections	Δρ_max_ = 1.43 e Å^−^^3^
191 parameters	Δρ_min_ = −0.89 e Å^−^^3^
0 restraints	

### Special details

**Table:**

Geometry. All esds (except the esd in the dihedral angle between two l.s. planes) are estimated using the full covariance matrix. The cell esds are taken into account individually in the estimation of esds in distances, angles and torsion angles; correlations between esds in cell parameters are only used when they are defined by crystal symmetry. An approximate (isotropic) treatment of cell esds is used for estimating esds involving l.s. planes.

### Fractional atomic coordinates and isotropic or equivalent isotropic displacement parameters (Å^2^)

**Table:**

	*x*	*y*	*z*	*U*_iso_*/*U*_eq_	
Zn1	−0.15526 (6)	0.48844 (6)	0.17897 (5)	0.02409 (17)	
Zn2	0.24043 (6)	0.62310 (6)	0.40311 (5)	0.02507 (18)	
P1	0.12600 (13)	0.63180 (14)	0.10765 (11)	0.0231 (3)	
H1	0.096928	0.778451	0.046688	0.028*	
P2	0.57579 (14)	0.67732 (15)	0.38453 (12)	0.0249 (3)	
H2	0.585406	0.817529	0.343455	0.030*	
P3	−0.05587 (14)	0.79747 (15)	0.60236 (12)	0.0268 (3)	
H3	−0.092835	0.950694	0.595471	0.032*	
O1	0.2488 (4)	0.5969 (4)	0.2136 (3)	0.0339 (8)	
O2	0.2100 (4)	0.5268 (4)	−0.0006 (3)	0.0354 (8)	
O3	−0.0360 (4)	0.6260 (4)	0.1659 (4)	0.0336 (8)	
O4	0.6946 (4)	0.5953 (4)	0.4998 (3)	0.0292 (7)	
O5	0.6265 (4)	0.5929 (4)	0.2609 (3)	0.0347 (8)	
O6	0.3994 (4)	0.7076 (4)	0.4335 (3)	0.0296 (7)	
O7	0.0241 (4)	0.7586 (4)	0.4650 (3)	0.0374 (8)	
O8	0.0481 (4)	0.7208 (4)	0.7225 (4)	0.0386 (8)	
O9	−0.2216 (4)	0.7747 (5)	0.6275 (4)	0.0495 (10)	
H1O	−0.248833	0.714280	0.588686	0.059*	
C1	0.5538 (6)	0.2241 (8)	0.0263 (6)	0.0480 (15)	
H1A	0.501366	0.270784	−0.057545	0.058*	
C2	0.6748 (6)	0.0729 (7)	0.0373 (5)	0.0398 (13)	
C3	0.7424 (6)	0.0139 (6)	0.1628 (5)	0.0365 (12)	
H3A	0.822779	−0.090250	0.177280	0.044*	
C4	0.6958 (7)	0.1029 (7)	0.2676 (5)	0.0424 (13)	
H4	0.745714	0.061680	0.352982	0.051*	
C5	0.5791 (7)	0.2484 (7)	0.2484 (6)	0.0462 (15)	
H5	0.546317	0.311069	0.320035	0.055*	
C6	0.7301 (10)	−0.0189 (10)	−0.0812 (7)	0.076 (2)	
H6A	0.645693	0.024450	−0.148650	0.113*	
H6B	0.747583	−0.130136	−0.047771	0.113*	
H6C	0.832992	−0.010706	−0.124466	0.113*	
N1	0.5110 (5)	0.3034 (5)	0.1310 (6)	0.0501 (13)	
H1B	0.432689	0.397922	0.121074	0.060*	

### Atomic displacement parameters (Å^2^)

**Table:**

	*U*^11^	*U*^22^	*U*^33^	*U*^12^	*U*^13^	*U*^23^
Zn1	0.0207 (3)	0.0390 (3)	0.0137 (3)	−0.0117 (2)	0.0011 (2)	−0.0071 (2)
Zn2	0.0217 (3)	0.0384 (3)	0.0167 (3)	−0.0109 (2)	0.0002 (2)	−0.0094 (2)
P1	0.0208 (6)	0.0313 (6)	0.0170 (6)	−0.0083 (5)	−0.0011 (4)	−0.0064 (5)
P2	0.0231 (6)	0.0367 (7)	0.0183 (6)	−0.0141 (5)	−0.0002 (5)	−0.0067 (5)
P3	0.0226 (6)	0.0356 (7)	0.0231 (6)	−0.0106 (5)	0.0001 (5)	−0.0079 (5)
O1	0.0318 (18)	0.056 (2)	0.0185 (17)	−0.0173 (16)	−0.0047 (14)	−0.0113 (15)
O2	0.0284 (17)	0.058 (2)	0.0178 (17)	−0.0078 (16)	−0.0033 (14)	−0.0176 (16)
O3	0.0247 (17)	0.0344 (18)	0.044 (2)	−0.0114 (15)	0.0067 (15)	−0.0161 (16)
O4	0.0279 (17)	0.0387 (19)	0.0237 (17)	−0.0145 (15)	−0.0031 (14)	−0.0055 (14)
O5	0.0205 (16)	0.066 (2)	0.0187 (16)	−0.0148 (16)	0.0025 (13)	−0.0153 (16)
O6	0.0215 (16)	0.046 (2)	0.0266 (17)	−0.0132 (15)	0.0041 (13)	−0.0192 (15)
O7	0.0236 (17)	0.054 (2)	0.0286 (19)	−0.0059 (16)	0.0002 (14)	−0.0126 (16)
O8	0.045 (2)	0.043 (2)	0.0290 (19)	−0.0170 (17)	−0.0059 (16)	−0.0032 (16)
O9	0.034 (2)	0.080 (3)	0.052 (2)	−0.033 (2)	0.0135 (17)	−0.038 (2)
C1	0.026 (3)	0.069 (4)	0.040 (3)	−0.017 (3)	−0.007 (2)	0.016 (3)
C2	0.036 (3)	0.057 (4)	0.033 (3)	−0.025 (3)	0.008 (2)	−0.014 (3)
C3	0.025 (2)	0.033 (3)	0.043 (3)	−0.007 (2)	0.000 (2)	0.005 (2)
C4	0.046 (3)	0.057 (4)	0.029 (3)	−0.029 (3)	−0.004 (2)	0.005 (3)
C5	0.054 (4)	0.052 (4)	0.044 (4)	−0.034 (3)	0.029 (3)	−0.021 (3)
C6	0.088 (5)	0.115 (6)	0.053 (4)	−0.062 (5)	0.024 (4)	−0.047 (4)
N1	0.030 (2)	0.033 (2)	0.066 (4)	0.000 (2)	0.024 (2)	−0.001 (2)

### Geometric parameters (Å, º)

**Table:**

Zn1—O3	1.923 (3)	P3—O9	1.543 (3)
Zn1—O8^i^	1.930 (4)	P3—H3	1.3200
Zn1—O2^ii^	1.939 (3)	O9—H1O	0.8542
Zn1—O5^iii^	1.945 (3)	C1—N1	1.318 (8)
Zn2—O6	1.931 (3)	C1—C2	1.392 (8)
Zn2—O1	1.931 (3)	C1—H1A	0.9500
Zn2—O7	1.938 (3)	C2—C3	1.374 (8)
Zn2—O4^iv^	1.967 (3)	C2—C6	1.504 (8)
P1—O3	1.493 (3)	C3—C4	1.375 (8)
P1—O1	1.511 (3)	C3—H3A	0.9500
P1—O2	1.513 (3)	C4—C5	1.341 (8)
P1—H1	1.3200	C4—H4	0.9500
P2—O6	1.507 (3)	C5—N1	1.304 (8)
P2—O5	1.509 (3)	C5—H5	0.9500
P2—O4	1.533 (3)	C6—H6A	0.9800
P2—H2	1.3200	C6—H6B	0.9800
P3—O8	1.492 (4)	C6—H6C	0.9800
P3—O7	1.496 (3)	N1—H1B	0.8800
			
O3—Zn1—O8^i^	114.37 (14)	P1—O3—Zn1	138.2 (2)
O3—Zn1—O2^ii^	112.19 (15)	P2—O4—Zn2^iv^	128.89 (18)
O8^i^—Zn1—O2^ii^	109.65 (15)	P2—O5—Zn1^v^	129.60 (18)
O3—Zn1—O5^iii^	107.88 (14)	P2—O6—Zn2	134.27 (19)
O8^i^—Zn1—O5^iii^	111.45 (15)	P3—O7—Zn2	134.0 (2)
O2^ii^—Zn1—O5^iii^	100.45 (13)	P3—O8—Zn1^i^	138.6 (2)
O6—Zn2—O1	111.84 (14)	P3—O9—H1O	125.5
O6—Zn2—O7	108.97 (14)	N1—C1—C2	120.7 (5)
O1—Zn2—O7	112.00 (13)	N1—C1—H1A	119.6
O6—Zn2—O4^iv^	108.32 (13)	C2—C1—H1A	119.6
O1—Zn2—O4^iv^	102.86 (14)	C3—C2—C1	115.7 (5)
O7—Zn2—O4^iv^	112.73 (14)	C3—C2—C6	122.1 (6)
O3—P1—O1	114.3 (2)	C1—C2—C6	122.1 (6)
O3—P1—O2	115.04 (19)	C2—C3—C4	121.2 (5)
O1—P1—O2	107.52 (19)	C2—C3—H3A	119.4
O3—P1—H1	106.4	C4—C3—H3A	119.4
O1—P1—H1	106.4	C5—C4—C3	119.5 (5)
O2—P1—H1	106.4	C5—C4—H4	120.3
O6—P2—O5	112.30 (17)	C3—C4—H4	120.3
O6—P2—O4	112.74 (18)	N1—C5—C4	119.7 (5)
O5—P2—O4	112.38 (19)	N1—C5—H5	120.2
O6—P2—H2	106.3	C4—C5—H5	120.2
O5—P2—H2	106.3	C2—C6—H6A	109.5
O4—P2—H2	106.3	C2—C6—H6B	109.5
O8—P3—O7	116.5 (2)	H6A—C6—H6B	109.5
O8—P3—O9	111.5 (2)	C2—C6—H6C	109.5
O7—P3—O9	111.4 (2)	H6A—C6—H6C	109.5
O8—P3—H3	105.5	H6B—C6—H6C	109.5
O7—P3—H3	105.5	C5—N1—C1	123.2 (5)
O9—P3—H3	105.5	C5—N1—H1B	118.4
P1—O1—Zn2	136.5 (2)	C1—N1—H1B	118.4
P1—O2—Zn1^ii^	135.1 (2)		

Symmetry codes: (i) −*x*, −*y*+1, −*z*+1; (ii) −*x*, −*y*+1, −*z*; (iii) *x*−1, *y*, *z*; (iv) −*x*+1, −*y*+1, −*z*+1; (v) *x*+1, *y*, *z*.

### Hydrogen-bond geometry (Å, º)

**Table:**

*D*—H···*A*	*D*—H	H···*A*	*D*···*A*	*D*—H···*A*
N1—H1*B*···O1	0.88	2.20	2.992 (6)	150
N1—H1*B*···O2	0.88	2.24	2.947 (6)	138
O9—H1*O*···O4^iii^	0.85	1.78	2.630 (4)	177
C1—H1*A*···O5^vi^	0.95	2.34	3.285 (6)	175
C5—H5···O4^iv^	0.95	2.59	3.368 (6)	140
C5—H5···O6^iv^	0.95	2.52	3.300 (7)	140

Symmetry codes: (iii) *x*−1, *y*, *z*; (iv) −*x*+1, −*y*+1, −*z*+1; (vi) −*x*+1, −*y*+1, −*z*.
